# Carborane-Based Carbonic Anhydrase Inhibitors: Insight into CAII/CAIX Specificity from a High-Resolution Crystal Structure, Modeling, and Quantum Chemical Calculations

**DOI:** 10.1155/2014/389869

**Published:** 2014-09-18

**Authors:** Pavel Mader, Adam Pecina, Petr Cígler, Martin Lepšík, Václav Šícha, Pavel Hobza, Bohumír Grüner, Jindřich Fanfrlík, Jiří Brynda, Pavlína Řezáčová

**Affiliations:** ^1^Institute of Molecular Genetics, Academy of Sciences of the Czech Republic, Vídeňská 1083, 140 00 Prague 4, Czech Republic; ^2^Structural Genomics Consortium, University of Toronto, Toronto, ON, Canada M5G 1L7; ^3^Institute of Organic Chemistry and Biochemistry, Academy of Sciences of the Czech Republic, Gilead Sciences and IOCB Research Center, Flemingovo nam. 2, 166 10 Prague 6, Czech Republic; ^4^Institute of Inorganic Chemistry, Academy of Sciences of the Czech Republic, v.v.i., Hlavní 1001, 250 68 Řež near Prague, Czech Republic; ^5^Regional Center of Advanced Technologies and Materials, Department of Physical Chemistry, Palacký University, 77146 Olomouc, Czech Republic

## Abstract

Carborane-based compounds are promising lead structures for development of inhibitors of carbonic anhydrases (CAs). Here, we report structural and computational analysis applicable to structure-based design of carborane compounds with selectivity toward the cancer-specific CAIX isoenzyme. We determined the crystal structure of CAII in complex with 1-methylenesulfamide-1,2-dicarba-*closo*-dodecaborane at 1.0 Å resolution and used this structure to model the 1-methylenesulfamide-1,2-dicarba-*closo*-dodecaborane interactions with CAIX. A virtual glycine scan revealed the contributions of individual residues to the energy of binding of 1-methylenesulfamide-1,2-dicarba-*closo*-dodecaborane to CAII and CAIX, respectively.

## 1. Introduction

Carbonic anhydrases (CAs) play important roles in many physiological and pathophysiological processes. For example, extracellular CAs participate in tumor growth and progression [1]. CAIX, which is selectively expressed in a range of hypoxic tumors, is a validated diagnostic and therapeutic target (recently reviewed in [2–4]). There are 15 human CA isoenzymes, and due to the ubiquity of these enzymes in human tissues, selective inhibition is a very important aspect of drug design.

Three main classes of CA inhibitors have been described to date (reviewed in [5]): (i) metal ion binders (sulfonamides, sulfamides, sulfamates, dithiocarbamates, thiols, and hydroxamates); (ii) compounds that anchor the zinc-coordinated water molecule/hydroxide ion (phenols, carboxylates, polyamines, esters, and sulfocoumarins); and (iii) coumarins and related compounds that bind further away from the metal ion.

CA inhibitors from the first class (metal ion binders) contain specific functional groups that interact with the catalytic Zn^2+^ ion in the CA active site. These metal-binding functionalities are typically joined to a “ring” structure. This moiety is not necessarily aromatic; however, it is usually consisting of a 5- or 6-membered hydrocarbon ring or conjugated ring system containing nitrogen, oxygen, and/or sulfur. Numerous functional groups have been added to the ring structure scaffold to modify inhibitor properties such as specificity toward a particular CA isoenzyme, pK_a_, or solubility (reviewed in [6]). Recently, we reported design of CA inhibitors containing space-filling carborane clusters in place of the typical ring structure [7]. We showed that various carborane clusters act as CA inhibitors and that modifying these clusters with an appropriately attached sulfamide group and other substituents leads to compounds with selectivity toward the cancer-specific CAIX isoenzyme.

Boron is one of few chemical elements that can form binary hydrides composed of more than two atoms, which leads to formation of boron cluster compounds (boron hydrides or boranes). Their basic structural feature is formation of a polyhedron with triangular facets held together by 3-center 2-electron bonds with an extensive electron delocalization [8]. A typical structural archetype is represented by the divalent* closo*-B_12_H_12_^2−^ anion, an extremely stable compound with a symmetrical 12-vertex icosahedron structure [9]. Replacement of one or more {BH^−^} in borane cage with {CH} leads to series of carboranes and removal of BH vertices leads to various open-cage (*nido*-) species. Carboranes thus offer a large variety of structural archetypes that provide interesting counterparts to organic compounds [10].

Many features of icosahedral 12-vertex carboranes are useful in the design of biologically active compounds. Carboranes have high thermal and chemical stability; therefore, they generally do not undergo catabolism and are nontoxic to the host organism [11, 12]. The basic* closo-*C_2_B_10_H_12_ carborane cluster is highly hydrophobic [13]; however, its controlled deboronation can generate water soluble 11-vertex* nido*-C_2_B_9_H_12_^−^. These anions represent important intermediates in the synthesis of a family of mainly anionic metal bis(dicarbollides) accessible via metal insertion. Incorporation of carborane cages into the structures of certain substances of medicinal interest can enhance hydrophobic interactions between the boron cluster-coupled pharmaceuticals and their protein targets, increase* in vivo* stability, and facilitate uptake through cellular membranes [14, 15]. The successful use of boron clusters as hydrophobic pharmacophores has recently been increasing [16, 17]. Examples of carborane pharmacophores include boron-containing antifolates [18], HIV protease inhibitors [19, 20], and estrogen receptor agonists and antagonists [21], among others [16, 22, 23].

Drug design efforts benefit greatly from knowledge of the 3D structures of protein-ligand complexes. X-ray crystallography has contributed considerably to the development of CA inhibitors; more than 500 structures of human CA isoenzymes (wild-type and mutant forms) in complex with various inhibitors have offered unprecedented insight into inhibitor binding modes (reviewed in [24]). Structural information coupled with experimental inhibition data can be used to validate various computational approaches to assess inhibitor binding strength. Once a particular theoretical approach reproduces the known data well, it can be used for prospective design. For studies involving metal ions and unusual compounds such as boranes, the use of quantum chemistry (QM) is warranted [25, 26]. Indeed, we recently used a quantum mechanics/molecular mechanics (QM/MM) methodology to quantitatively describe the binding of two carborane-based sulfamides to CAII [7] and to explain fundamental differences in the binding modes of* closo*- and* nido*-cages [27].

**Figure 1:**
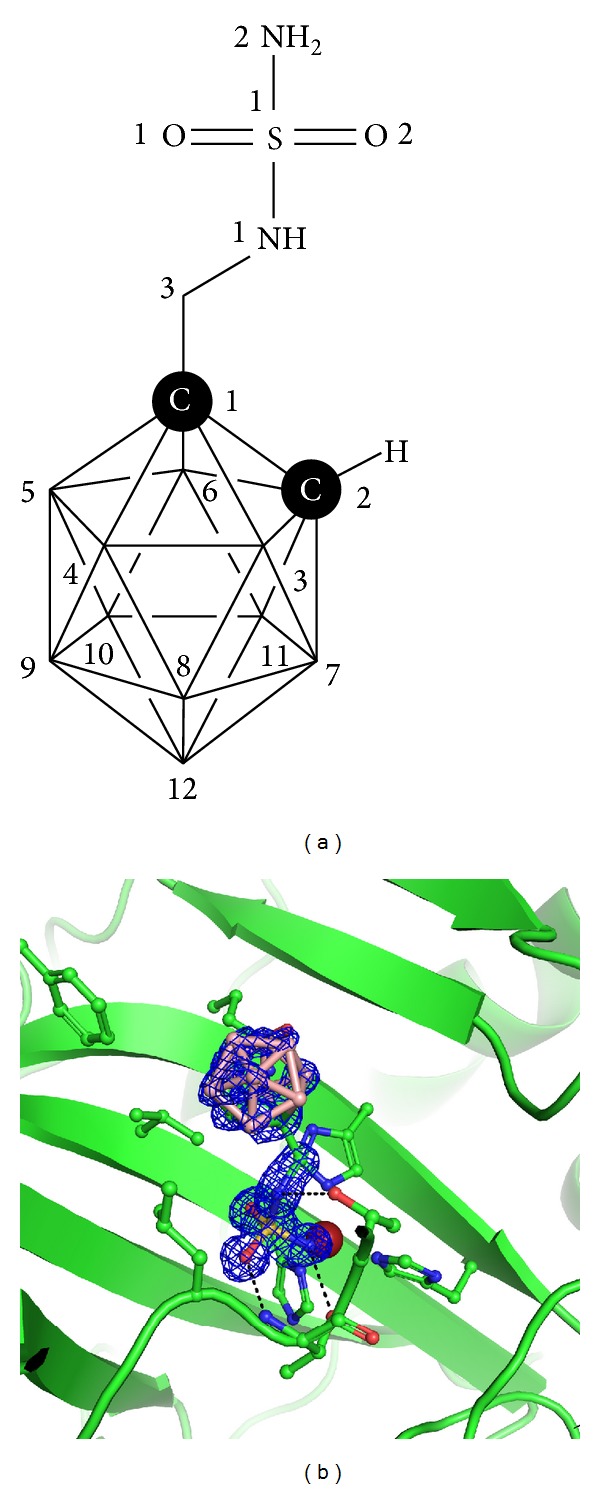
(a) Structural formula of** 1** with atom numbers used in the crystal structure coordinate file. The vertices in carborane cluster represent BH groups. (b) Crystal structure of CAII in complex with** 1**. The CAII active site is shown in cartoon representation; residues involved in interactions with the Zn^2+^ ion (purple sphere) and** 1** are shown in stick representation with carbon atoms colored green. Boron atoms are colored pink, and other heteroatoms are colored according to standard color coding: oxygen, red; nitrogen, blue; sulfur, yellow. The 2*Fo-Fc* electron density map for** 1** is contoured at 1 σ.

Here, we report the X-ray structure of CAII with bound 1-methylenesulfamide-1,2-dicarba-*closo*-dodecaborane (compound** 1**, Figure 1(a)) determined at 1.0 Å resolution. This atomic-level resolution allowed us to assess in detail the positions of carbon and boron atoms in the carborane cage of** 1**. Additionally, we modeled the complex of** 1** with CAIX. We employed a virtual glycine scan to analyze the differences between the interactions of** 1** with CAII and CAIX.

## 2. Materials and Methods

### 2.1. Protein Crystallization and Diffraction Data Collection

For crystallization of human CAII (Sigma, catalogue number C6165) in complex with 1-methylenesulfamide-1,2-dicarba-*closo*-dodecaborane (compound** 1**), we adapted a previously described procedure [28]. CAII (at a concentration of 4 mg*·*mL^−1^, dissolved in water) was incubated in aqueous solution containing a 2-fold molar excess of* p*-hydroxymercuribenzoate (Sigma, catalogue number 55540). The protein was concentrated to 10 mg*·*mL^−1^ and unbound* p*-hydroxymercuribenzoate was removed with Amicon Ultra-4 concentrators (Merck-Millipore MWCO 10 kDa).

The complex of CAII with** 1** was prepared by adding a 1.1-fold molar excess of** 1 **(in DMSO) to the 10 mg*·*mL^−1^ solution of CAII in water without pH adjustment (the final DMSO concentration did not exceed 5% v/v).

The best diffracting crystals were obtained using the hanging-drop vapor diffusion method under the following conditions: 2 *μ*L protein-inhibitor complex solution was mixed with 2 *μ*L precipitant solution [2.5 M (NH_4_)_2_SO_4_, 0.3 M NaCl, and 100 mM Tris-HCl, pH 8.2] and equilibrated over a reservoir containing 1 mL of precipitant solution at 18°C. Crystals with dimensions of 0.3 mm × 0.1 mm × 0.1 mm grew within 7 days.

For cryoprotection, the crystals were incubated in mother liquor supplemented with 25% glycerol for approximately 30 s, flash-frozen, and stored in liquid nitrogen. Diffraction data for the CAII complex were collected at 100 K at the X14.2 BESSY beamline in Berlin, Germany [29]. Data were collected in two passes: the high-resolution range (11.75–1.00 Å) and the low-resolution range (21.08–1.20 Å). The two datasets were integrated with iMOSFLM [30] and merged and scaled with SCALA [31]. Data collection and refinement statistics are summarized in Table 1.

### 2.2. Structure Determination, Refinement, and Analysis

Crystal structures were solved by difference Fourier method using the CAII structure (PDB code 3IGP [34]) as a starting model. The model was refined using REFMAC5 [35], part of the CCP4 program suite [36]. The model was initially refined with isotropic atomic displacement parameters (ADPs); hydrogen atoms in riding positions were added later. For the final rounds of refinement, we used a mixed isotropic-anisotropic model of ADPs: anisotropic ADPs were used for all atoms, and only atoms in alternative conformations were refined isotropically. Atomic coordinates for the structure of** 1** were generated by quantum mechanics computation with DFT-D methodology [37] using the B-LYP functional and SVP basis set [38] in the Turbomole program [39]. A geometric library for** 1** was generated using the Libcheck program from the CCP4 suite. Coot [40] was used for rebuilding. The quality of the refined model was assessed using MolProbity [33]. The coordinates and structure factors were deposited in the PDB under accession code 4Q78. Final refinement statistics are summarized in Table 1. All structural figures were prepared using PyMOL 1.4.1 [41].

### 2.3. Model of CAIX-1 Complex

The complex of CAIX and** 1** was modeled by aligning the existing crystal structures of the CAIX catalytic domain (PDB code 3IAI [42]) with the CAII-**1** complex (PDB code 4MDG [7]) using PyMOL version 1.2 [43]. Preparation of structure coordinate files for further calculations was performed as described before for CAII [27].

The complex was fully optimized using a QM/MM procedure. We used ONIOM-like subtractive scheme [44] with link atoms and mechanical embedding to be consistent with our previous studies [27, 45–48]. The QM part is described at the DFT-D TPSS/TZVP//BLYP/SVP level of theory [39] and comprises 218 atoms including the atoms present in** 1** and 8 amino acids (Trp5, Asn62, His64, Gln67, Gln92, Val131, Leu135, and Pro202). The MM part constituted the remainder of the protein, and the surrounding solvent was approximated by a generalized Born (GB) implicit model. Detailed description of the procedure was published in [27]. One crystal water molecule (Wat272) bridging the inhibitors and CAII residues Thr199, Glu106, and Tyr7 was retained to maintain the integrity of the active site. Other water molecules present in the crystal structures were omitted.

The positions of the added hydrogen atoms,** 1**, and 15 amino acids surrounding the ligand (Trp5, Asn62, Gly63, His64, Gln67, Leu91, Gln92, Leu123, Val131, Leu135, Leu141, Thr200, Pro201, Pro202, and Ala204) were relaxed in a GB implicit solvent model using the FIRE algorithm followed by 10 ps annealing from 100 K or 150 K to 0 K using the Berendsen thermostat [49] in the SANDER module of the AMBER 10 package [50].

### 2.4. Virtual Glycine Scan

The contribution of the active site amino acids to inhibitor binding was examined by virtual glycine scanning. Individual amino acids in contact with** 1** in the CAIX-**1** model and CAII-**1** crystal structure were substituted with glycine. The energy contributions (ΔΔG_int⁡_′) were calculated as the difference between the original ΔG_int⁡_′ at the QM/MM level with the wild-type amino acid and the new ΔG_int⁡_′ with the mutated glycine residue [27].

## 3. Results and Discussion

### 3.1. Crystal Structure of CAII in Complex with **1** at Atomic Resolution

The overall structure of CAII in complex with** 1** was refined to 1.0 Å resolution. This high resolution allowed us to observe details that could not be fully resolved in the complex structure determined previously at lower resolution. Atomic resolution was achieved by derivatization of CAII using the 4-(hydroxymercury)benzoic acid (abbreviated MBO in the cif library of small molecules) method described by [28]. The mercury atom of MBO covalently binds to S*γ* of Cys206. This modification allows formation of a hydrogen bond between the OZ1 oxygen of the MBO carboxyl group and the main-chain amino group of Tyr40 in the neighboring protein molecule, reinforcing the crystal lattice and increasing the diffraction quality of the crystal. In our structure, MBO is modeled in two alternative conformations with occupancies of 0.6 and 0.2.

When our atomic resolution structure is compared with the structure of the CAII-**1** complex determined at 1.35 Å resolution (PDB code 4MDG [7]), the RMSD value for superposition of the C*α* atoms of residues 4–261 is 0.142 Å, a value typical for superposition of identical structures [51]. The N-terminal residue His3 is traced differently in the two structures; double conformations of numerous side chains (e.g., Glu14, His64, and Gln74) are resolved in the atomic resolution structure. We found an additional difference in the loop formed by amino acid residues 124–139, with a maximum difference of 0.738 Å for the position of Gln136 C*α*. Gln136 forms van der Waals contacts with the MBO covalently attached to Cys206. The positions of Phe131 and Val135, which form a hydrophobic rim at the active site, are also influenced by MBO binding. This results in a subtle positional shift of the inhibitor, with an RMSD of 0.145 Å for superposition of 12 atoms in the carborane cage of** 1** bound to CAII and CAII derivatized by MBO. This value is below the value observed for superposition of identical structures [51].

Atomic-level resolution allowed us to resolve the carbon and boron atom positions in the symmetrical carborane cage of** 1**. When analyzing the values of the electron density map at positions of atoms bonded to the C1 atom, we can assume that positions with higher density levels are more likely to be carbon than boron atoms. Similar analysis was done by others for boron-containing inhibitor of human dihydrofolate reductase [18]. The C2 atom of the carborane cage (Figure 1(a)) was modeled into the position with an electron density value of 1.16 e/Å^3^, which was approximately 0.15 e/Å^3^ higher than those for the B3, B4, B5, and B6 atoms. To exclude the possibility that higher density is caused by model bias, we altered the composition of the cage by replacing the C2 atom with a boron atom. Electron density values did not change significantly after several rounds of refinement cycles.

Thus, we can conclude that the most probable position of the second carbon atom in the carborane cage of** 1** is the position assigned to the C2 atom in our crystal structure. This is in good agreement with the recently published QM/MM modeling study [27].

### 3.2. Detailed Analysis of Inhibitor Interactions with CAII

**Figure 2:**
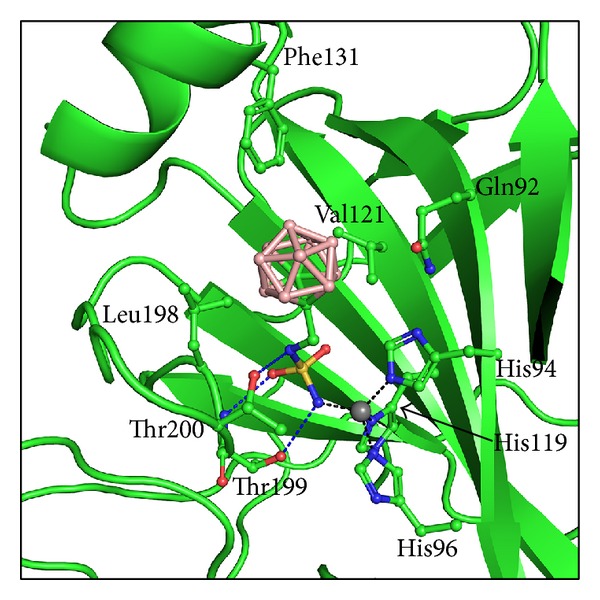
Interactions of** 1** with CAII. The protein is shown in cartoon representation; residues involved in interactions with the Zn^2+^ ion (gray sphere) and** 1** are shown in stick representation. Polar interactions are represented by blue dashed lines; Zn^2+^ ion coordination is shown as black dashed lines.

The crystal structure of human CAII in complex with** 1** determined at 1.0 Å resolution confirmed the key interactions that our group observed previously [7]. The compound fits very well into the CAII active site cavity and makes numerous polar and nonpolar interactions with the residues in the enzyme active site. The sulfamide moiety, which forms key polar interactions with the active site Zn^2+^ ion, also makes polar interactions with Thr199 typical of other sulfamide inhibitors of CAII (Figure 2). The linker NH group forms an additional polar interaction with O*γ* of Thr200. The compound makes several van der Waals interactions with residues Gln92, His94, His96, His119, Val121, Phe131, Leu198, and Thr200 (Figure 2). All interactions between the inhibitor and protein are summarized in Table 2.

**Figure 3:**
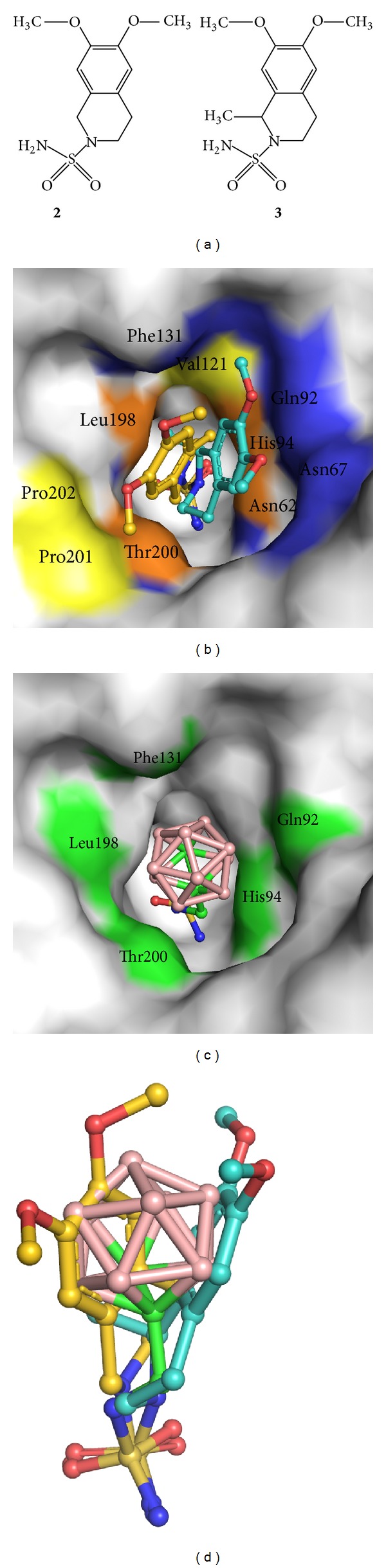
(a) Structural formulas of** 2** and** 3**. (b) Interactions of** 2** and** 3** with the CAII active site. Compound** 2** is represented with golden carbon atoms, while the carbon atoms of** 3** are colored turquoise. Surface of residues making contacts with the isoquinoline moiety of** 2** and** 3** are highlighted in yellow and blue, respectively. Surface of residues colored orange make contacts with both compounds. Atoms involved in contacts with the sulfonamide groups are not highlighted. (c) Interactions of** 1** with the CAII active site. Surface of residues making contacts with the carborane and linker moiety of** 1** are highlighted in green. Atoms involved in contacts with the sulfonamide groups are not highlighted. (d) Superposition of binding poses of** 1**,** 2**, and** 3** in the CAII active site. Superposition of the complex structures was based on the best fit for C*α* atoms of CAII residues 6–261.

The idea of designing CA inhibitors containing a carborane cluster moiety originated from our previous structural studies of isoquinoline-containing sulfonamide inhibitors (Figure 3(a)). Structural analysis of CAII in complex with 6,7-dimethoxy-1,2,3,4-tetrahydroisoquinolin-2-ylsulfonamide (**2**, PDB code 3IGP, [34]) and 6,7-dimethoxy-1-methyl-1,2,3,4-tetrahydroisoquinolin-2-ylsulfonamide (**3**, PDB code 3PO6, [52]) revealed two distinct binding modes that engage two opposite sides of the enzyme active site cavity (Figure 3(b)). Following this analysis, we hypothesized that the binding space within the enzyme active site cavity could be effectively filled with a bulky hydrophobic molecule with a spherical structure. This led to design of** 1** which exhibited inhibitory property to CAII and CAIX with Ki values in submicromolar range. Structural analysis of CAII-**1** indicates that our structure-based design was sound. We found that the carborane cluster interacts with both sides of the enzyme active site as predicted (Figure 3(c), Table 3) and that the position of** 1** in the CAII active site superposes well with the two binding modes observed for 2 and 3 (Figure 3(d)).

### 3.3. Model of the CAIX-1 Complex

**Figure 4:**
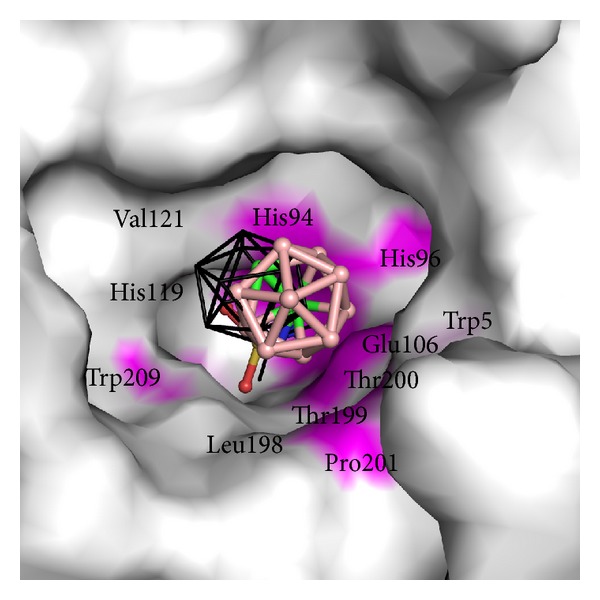
Interactions of** 1** with the CAIX active site. Atoms making contacts with the carborane and linker moiety of** 1** are highlighted in magenta. Atoms involved in contacts with the sulfonamide groups are not highlighted. Superposition of the binding pose of** 1** in CAII is shown as black lines. Superposition is based on the best fit for C*α* atoms of all residues of CAII onto CAIX.

The CAII-**1** crystal structure was used to model binding of compound** 1 **into the CAIX active site using QM/MM methods (Figure 4).

The substrate binding sites of CAII and CAIX differ by only six amino acids: Asn67 of CAII is replaced by Gln in CAIX, Ile91 by Leu, Trp123 by Leu, Phe131 by Val, Val135 by Leu, and Leu204 by Ala. These variations result in a differently shaped active site cavity, which accommodated** 1** in a slightly different pose (Figure 4). While the position of the sulfamide anchor remained unchanged, the carborane cluster shifted by 2.1 Å (expressed as a difference in the position of B12) away from the central *β*-sheet. In CAIX-**1**, the carborane interacts more with the opposite site of the active site, specifically with amino acid residues His94, His96, Glu106, Leu198, Thr199, Thr200, and Pro201 (Figure 4, Table 3). All polar and van der Waals interactions between CAIX and** 1** are summarized in Table 4.

**Figure 5:**
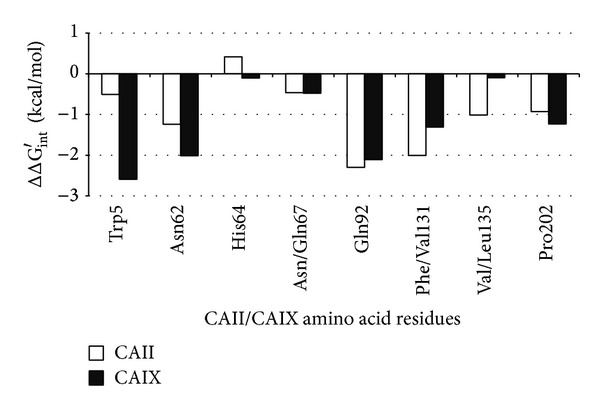
Results of virtual glycine scan showing contributions of individual residues to the energy of binding of** 1** to CAII and CAIX, respectively.

We used a virtual glycine scan to study the roles of individual amino acid side chains in the active sites of CAII and CAIX in binding of** 1**. The changes in free energy of interaction (ΔΔG_int⁡_′) upon mutation of a given amino acid residue to glycine are shown in Figure 5.

The largest energy change (2.6 kcal/mol) occurred for Trp5, which is positioned closer to** 1** in CAIX-**1** than in CAII-**1**. The side chain of Trp5 forms several dihydrogen bonds with the carborane cage of** 1**. The shortest one has a H ⋯ H distance of 2.3 Å. The other major contributor to strong CAIX-**1** binding was Asn62; the energy of binding exceeded that in CAII-**1** by nearly 1 kcal/mol. These contributions were cancelled out by differences in binding energy contributions of amino acid residues 131 (Phe/Val) and 135 (Val/Leu), which were lower in CAIX by 0.7 and 0.9 kcal/mol, respectively. The energy changes of other residues were small.

When we compared binding of** 1** to CAII and CAIX, we noted that the favorable energy changes in CAIX-**1** due to the binding of residues Trp5, Asn62, and His64 were slightly larger than the unfavorable changes in binding caused by the different amino acids at residues 131 and 135. This is in qualitative agreement with the experimental *K*_*i*_ values, which are 700 ± 141 nm for inhibition of CAII and 380 ± 111 nM for inhibition of CAIX [7].

## 4. Conclusions

We determined to atomic resolution the crystal structure of CAII in complex with 1-methylenesulfamide-1,2-dicarba-*closo*-dodecaborane (**1**), a parent compound of a recently reported series of CA inhibitors containing carborane cages [7]. Comparing this crystal structure with those of CAII complexes with conventional organic inhibitors showed that the three-dimensional cluster fills the enzyme active site cavity. Atomic-level resolution allowed us to distinguish the positions of carbon and boron atoms in the carborane cage. The crystal structure also served as a model for construction of the CAIX-**1 **computational model. Virtual glycine scan enabled us to quantify the contributions of individual residues to the energy of binding of** 1** to CAII and CAIX and uncover differences of the enzyme active site cavities. Structural and computational analysis will be used in future structure-based design of carborane compounds with selectivity toward the cancer-specific CAIX isoenzyme.

## Figures and Tables

**Table 1:** Data collection and refinement statistics.

Data collection statistics	
Space group	*P*2_1_
Cell parameters (Å; °)	42.20, 41.73, 72.16; 90.0, 104.4, 90.0
Wavelength (Å)	0.9184
Resolution (Å)	21.08–1.00 (1.05–1.00)
Number of unique reflections	108,781 (15,490)
Multiplicity	3.5 (2.5)
Completeness (%)	83.1 (81.4)
*R*_merge_^a^	0.056 (0.375)
Average *I*/*σ*(*I*)	10.8 (2.3)
Wilson B (Å^2^)	6.5
Refinement statistics	
Resolution range (Å)	69.90–1.00 (1.03–1.00)
No. of reflections in working set	97,856 (7,831)
No. of reflections in test set	5,426 (412)
*R* value (%)^b^	17.5 (24.4)
*R*_free_ value (%)^c^	20.0 (26.2)
RMSD bond length (Å)	0.011
RMSD angle (°)	1.53
Number of atoms in AU	2297
Number of protein atoms in AU	2081
Number of water molecules in AU	176
Mean ADP value protein/inhibitor (Å^2^)	12.0/17.6
Ramachandran plot statistics^d^	
Residues in favored regions (%)	96.56
Residues in allowed regions (%)	3.44

The data in parentheses refer to the highest-resolution shell.

^a^ *R*_merge_ = ∑_*hkl*_∑_*i*_ *I*_*i*_(*hkl*) − 〈*I*(*hkl*)〉∣/∑_*hkl*_∑_*i*_ *I*_*i*_(*hkl*), where *I*_*i*_(*hkl*) is the individual intensity of the *i*th observation of reflection *hkl* and 〈*I*(*hkl*)〉 is the average intensity of reflection *hkl* with summation over all data.

^b^ *R* value = ||*F*_*o*_ | − | *F*_*c*_ | |/|*F*_*o*_|, where *F*_*o*_ and *F*_*c*_ are the observed and calculated structure factors, respectively.

^c^ *R*_free_ is equivalent to *R* value but is calculated for 5% of reflections chosen at random and omitted from the refinement process [32].

^d^as determined by Molprobity [33].

**Table 2:** List of contacts between CAII and **1**.

CAII			**1**	
Residue		Atom	Atom^a^	Distance [Å]^b^
	**Zn**	**ZN**	**N2**	**1.87^c^**
	Zn	ZN	S	3.04
	Zn	ZN	O2	3.05
92	Gln	OE1	B6	3.47
92	Gln	OE1	B11	3.52
92	Gln	CD	B6	3.84
94	His	CE1	O2	2.97
94	His	NE2	N2	3.23
94	His	NE2	O2	3.31
94	His	CE1	C3	3.67
94	His	NE2	S	3.81
94	His	CE1	N2	3.82
94	His	CE1	S	3.84
94	His	NE2	C3	3.94
96	His	NE2	N2	3.14
96	His	CE1	N2	3.56
119	His	ND1	N2	3.39
119	His	ND1	O2	3.88
119	His	CE1	N2	3.96
121	Val	CG2	O2	3.82
131	Phe	CZ	B8	3.83
131	Phe	CZ	B7	3.97
198	Leu	CA	O1	3.09
198	Leu	C	O1	3.36
198	Leu	CB	O1	3.60
198	Leu	CD2	O1	3.63
198	Leu	CD1	B3	3.86
**199**	**Thr**	**N**	**O1**	**2.70**
**199**	**Thr**	**OG1**	**N2**	**2.74**
199	Thr	OG1	O1	3.58
199	Thr	OG1	S	3.78
199	Thr	N	S	3.83
199	Thr	CA	O1	3.83
199	Thr	CB	N2	3.98
**200**	**Thr**	**OG1**	**N1**	**3.02**
200	Thr	OG1	C3	3.14
200	Thr	OG1	B4	3.36
200	Thr	OG1	B3	3.56
200	Thr	OG1	C1	3.66

^a^Atom labels correspond to those shown in Figure 1(a).

^b^All contacts with a distance between ligand and protein (or Zn) atoms less than or equal to 4 Å are listed.

^c^Polar interactions are highlighted in bold.

**Table 3:** CAII or CAIX residues interacting with **1**, **2**, and **3**.

CAII			CAIX
**1^a^**	**2^b^**	**3^c^**	**1^d^**
			Trp5
		Asn62	
		**Asn67^e^**	
Gln92	Gln92	Gln92	
His94	His94	His94	His94
His96	His96	His96	His96
			Glu106
His119	His119	His119	His119
Val121		Val121	Val121
Phe131	Phe131	Phe131	
	Val143	Val143	
Leu198	Leu198	Leu198	Leu198
**Thr199**	**Thr199**	**Thr199**	**Thr199**
**Thr200**	Thr200	Thr200	**Thr200**
	Pro201		Pro201
	Pro202		
	Trp209		Trp209

Interacting residues were identified from ^a^crystal structure 4Q78 (this work); ^b^crystal structure 3IGP [34]; ^c^crystal structure 3PO6 [52]; ^d^computational model (this work); ^e^residues making polar interactions are highlighted in bold.

**Table 4:** Interactions between CAIX and **1**.

CAIX			**1**	
Residue		Atom	Atom^a^	Distance [Å]^b^
	**Zn**	**ZN**	**N2**	**2.1^c^**
	Zn	ZN	S	3.3
	Zn	ZN	O2	3.5
5	Trp	CZ2	B5	3.74
5	Trp	CZ2	B10	3.81
94	His	CE1	O2	3.15
94	His	CE1	C3	3.74
94	His	NE2	N2	3.36
94	His	NE2	S	3.88
94	His	NE2	O2	3.45
94	His	NE2	C3	3.76
96	His	CE1	N2	3.99
96	His	NE2	N2	3.49
106	Glu	OE2	N2	3.71
119	His	ND1	N2	3.37
119	His	CE1	N2	3.83
121	Val	CG2	O2	3.58
198	Leu	CA	O1	3.04
198	Leu	CB	O1	3.4
198	Leu	CD2	O1	3.43
198	Leu	C	O1	3.38
199	Thr	N	S	3.88
**199**	**Thr**	**N**	**O1**	**2.79**
199	Thr	CA	O1	3.96
199	Thr	CB	N2	3.85
**199**	**Thr**	**OG1**	**N2**	**2.63**
199	Thr	OG1	S	3.69
199	Thr	OG1	O1	3.65
200	Thr	OG1	C1	3.77
200	Thr	OG1	B5	3.56
**200**	**Thr**	**OG1**	**N1**	**3.13**
200	Thr	OG1	C3	3.31
200	Thr	OG1	B4	3.64
201	Pro	O	B4	3.6
201	Pro	O	B10	3.49
201	Pro	O	B8	3.96
209	Trp	CZ2	O1	3.74

^a^Atom labels correspond to those shown in Figure 1(a).

^b^All contacts with a distance less than or equal to 4 Å between ligand and protein (and Zn) atoms are listed.

^c^Polar interactions are highlighted in bold.
